# Supplementary material for: Complexome profiling on the Chlamydomonas lpa2 mutant reveals insights into PSII biogenesis and new PSII associated proteins
Source: J Exp Bot. 2021 Aug 26;73(1):245–62. doi: 10.1093/jxb/erab390 (PMC8730698; doi:10.1093/jxb/erab390)
Supplement: erab390_suppl_Supplementary_Dataset_S1 [file erab390_suppl_supplementary_dataset_s1.zip › Supplemental Dataset 1 - Excel List and all profiles/plots/YGG1_Cre02.g080250.html]

Trivial name: YGG1  
  
Euclidean distance: 10366.53  
Mean Intensity (WT): 669.99  
Mean Intensity (Mut): 348.46  
Distance: 15.47  
  
MapMan:   
  
p value of intensity sums Welch test: 0.3279
